# Twelve Drummers Drumming… With Dystonia

**DOI:** 10.5334/tohm.577

**Published:** 2021-02-08

**Authors:** Ian O. Bledsoe, Stephen G. Reich, Steven J. Frucht, Jennifer G. Goldman

**Affiliations:** 1 Weill Institute for Neurosciences, Department of Neurology, University of California, San Francisco, US; 2 Department of Neurology, University of Maryland School of Medicine, US; 3 Department of Neurology, New York University Langone Medical Center, US; 4 Department of Physical Medicine and Rehabilitation, Northwestern University Feinberg School of Medicine, US; 5 Department of Neurology, Northwestern University Feinberg School of Medicine, US; 6 Shirley Ryan AbilityLab, US

**Keywords:** musicians’ dystonia, drummers’ dystonia, focal hand dystonia, occupational dystonia, task specific dystonia

## Abstract

**Background:**

Reports of drummers’ dystonia are rare, particularly compared to the literature on dystonia in string, piano and brass players. Several cases of drummers’ dystonia have been included in large series of multiple instrumentalists, but there are few reports comprised exclusively of drummers with musicians’ dystonia. We present here a series of 12 drummers with task-specific, focal dystonia affecting their upper limbs while drumming and spanning multiple playing techniques and musical styles.

**Methods:**

We conducted a retrospective chart review of drummers with dystonia seen at academic Movement Disorders centers.

**Results:**

All 12 patients were male, and the majority eventually developed spread of dystonia to tasks other than drumming. Ten of the 12 had dystonia affecting their fingers, while 8/12 had dystonia affecting the wrist. Only 1/12 had involvement proximal to the wrist. Pharmacologic interventions were largely ineffective; 3 had some benefit from botulinum toxin injections, but this was limited by problematic weakness in one drummer.

**Discussion:**

The phenomenology in our series is concordant with prior reported cases, demonstrating frequent wrist involvement, though we also found that a greater proportion of patients had dystonia affecting the fingers. It could be hypothesized that different drumming techniques or musical styles modulate the relative risk of dystonic involvement of the different anatomical regions of the upper limb.

**Highlights:**

Drummers’ dystonia is one of the least common forms of musicians’ dystonia, though this may reflect fewer numbers of these instrumentalists. We present the largest series of drummers’ dystonia and review previously published cases. Our cohort, representing diverse drumming styles, showed frequent involvement of dystonia in the wrists and fingers.

## Introduction

Musicians’ dystonia is a particularly disabling form of occupational, task-specific focal hand dystonia and can be a career-ending disorder for professional musicians. It typically presents as a sustained twisting, tremor, or loss of coordination while playing an instrument. Musicians’ dystonia is estimated to affect 1% of professional musicians, but can affect amateurs and students as well [1]. The region of the body that is most commonly affected varies by instrument type but is often localized to the area of the body or limb producing the most rapid and highly skilled movements. This is reflected in more frequent involvement of the left hand in bowed string players, right hand in keyboard players, and embouchure in brass players [2].

Although drummers’ dystonia has been previously reported, it is relatively rare in the literature, particularly in comparison to reports in other types of musicians. Several cases affecting drummers have been included in large series of multiple instrumentalists, but there are few reports comprised exclusively of drummers. Out of all instrumentalists seen in performing arts clinics for focal dystonia, as reported in 4 large case series, only 1–5% were percussionists [3,4,5,6]. Drummers’ dystonia has been reported most frequently to affect the upper limbs, but recent case reports describe lower limb dystonia in drummers using pedals [7,8,9]. The largest published series of drummers’ dystonia to date included 6 drummers [6]. Here, we present a series of 12 drummers with focal hand dystonia seen in Movement Disorders clinics at academic medical centers and describe the clinical features of the drummers, the phenomenology of their dystonia, and compare with prior reports in the literature.

## Methods

This study was a retrospective chart review of drummers’ dystonia seen at academic medical centers (Columbia University Medical Center/Mount Sinai Health System [S.J.F.], Rush University Medical Center [J.G.G., I.O.B.], University of Maryland Medical Center [S.G.R.], University of California, San Francisco [I.O.B.]). The variables ascertained from the patient charts and videos were established *a priori* by the investigators. These variables included the age at which drumming was first started, instruments and musical styles played, age at development of dystonic symptoms, phenomenological features of dystonia, exacerbating and ameliorating factors, events prior to development of dystonic symptoms, and interventions tried. Descriptive statistics were used to describe the demographic and clinical features of the drummers in the cohort.

## Results

### Description of cohort (*TABLE 1*)

**Table 1:** Clinical characteristics of drummers with dystonia from multiple cohorts.


COHORT	SEX	RACE (CURRENT SERIES ONLY)	AGE AT ONSET, YEARS	AGE AT PRESENTATION/EVALUATION, YEARS	SIDE AFFECTED	DOMINANT HAND	INSTRUMENTS (MUSICAL STYLE)	PHENOMENOLOGY WITH INSTRUMENT	TREMOR REPORTED	SPREAD TO OTHER TASKS	BENEFICIAL INTERVENTIONS

Current series, No. 1 [Video, Case 1]	M	Caucasian	23	27	Left	Unknown	Drum, Xylophone	Ext 2, 3, 4, 5	Yes	Yes – typing	None (trial ofcarbidopa/levodopa without benefit, unknown dose)

Current series, No. 2 [Video, Case 2]	M	Caucasian	41	44	Right	Right	Drum set (Pop)	Flex 2, 3, 4, 5; wrist ulnar deviation	No	Yes – Golfing, brushing teeth, holding knife	None (lorazepam without benefit, unknown dose; referred for botulinum toxin injections but lost to follow up and results unknown)

Current series, No. 3 [Video, Case 3]	M	Caucasian	43	45	Right	Unknown	Indian tabla, classical percussion	Flex 2	No	No	External sensory trick with tape and orthopedic finger splint

Current series, No. 4 [Video, Case 4]	M	Caucasian	22	25	Left	Right	Classical Percussion	Irregular tremor at wrist; Flex 3, 4, 5 (PIP/DIP); Wrist Flex, radial deviation	Yes	No	Declined botulinum toxin injections or other treatment trials.

Current series, No. 5	M	Caucasian	53	54	Left	Right	Drums (jazz, rock)	Wrist Flex and ulnar deviation	Yes	Yes – putting on glasses, drinking from cup	None (multiple treatments tried including carbidopa/levodopa 600 mg daily, clonazepam, trihexyphenidyl 30 mg/daily, Botulinum toxin A injections, with EMG guidance – mild benefit, but limited by weakness: 25 units to each of left FCU, left pronator quadratus, left ECU; occupational therapy, limb immobilization)

Current series, No. 6	M	Caucasian	19	20	Both	Right	Drums (multiple genres)	Right: Flex 4, 5 (PIP); Left: Flex 4, 5 (PIP), wrist Ext and ulnar deviation	No	No	Good benefit with botulinum toxin A injections using EMG guidance – Right side: FCU 20 units, FDS 25 units. Left side: FCR 15 units, FCU 20 units, EDC 15 units. (Carbidopa/levodopa 450 mg daily with no benefit)

Current series, No. 7	M	Caucasian	33	42	Right	Right	Drums, xylophone, vibraphone (multiple genres)	Ext 2, abduction of 5; loosened thumb grip; tightness in forearm and wrist	No	No	Trihexyphenidyl 24 mg daily, modest benefit. Botulinum toxin A injections, with EMG guidance, 60–70% improvement: Right EPL 7.5 units; Right EIP 7.5 units; Right ECR 10 units

Current series, No. 8	M	Unknown	39	42	Right	Right	Indian tabla	Wrist ulnar deviation	No	No	Referred for botulinum toxin injections, but lost to follow up and results unknown

Current series, No. 9	M	Unknown	31	34	Left	Right	Classical percussion	Ext 4, 5 (at MCP), wrist Ext and radial deviation	No	Yes – holding stick between hands	None (Clonazepam, uncertain dose; trihexyphenidyl 6 mg daily without benefit; referred for botulinum toxin injections, but results unknown)

Current series, No. 10	M	Unknown	45	52	Left	Right	African-Cuban and African-Caribbean drumming	Ext 2, 3, 4 (at PIP, DIP); Flex 2, 3, 4 (at MCP); wrist Flex, shoulder elevation	Yes	Yes – handling fork, newspaper	None (Received 2 cycles of botulinum toxin injections elsewhere without benefit; unknown injection pattern)

Current series, No. 11	M	Unknown	39	48	Left	Unknown	Timpani (Classical)	Ext 3, 4, 5 (at PIP); Flex 3, 4, 5 (at MCP); abduction of digits; wrist ulnar deviation	No	No	Diazepam with modest benefit – unknown dose.

Current series, No. 12	M	Caucasian	34	38	Right	Right	Snare drum (Multiple genres)	Flex 2, 3, 4, tremor when writing	Yes	Yes – writing, brushing teeth	Propranolol LA 60 mg daily modest benefit; carbidopa/levodopa 250 mg daily without benefit.

Lederman 2004 [6]	M		34	35	Right	Left	Drum set (Jazz/Rock)	Right forearm supination, wrist Flex	No	Yes – in opposite hand	Trihexyphenidyl (modest)

Lederman 2004	M		21	22	Right	Left	Classical Percussion	Right forearm tightening, thumb slides off drumstick	No	No	None

Lederman 2004	M		36	39	Right	Right	Drum set (Country Music)	Right wrist Flex, thumb abduction/extension	No	Yes	None

Lederman 2004	M		22	23	Left	Right	Unspecified – Master’s Degree performance program	Left forearm supination and tremor	Yes	No	None

Lederman 2004	F		42	52	Both (Left > Right)	Right	Classical Percussion (Snare drum most affected; also xylophone and other mallet instruments)	Left wrist Ext, tremor; mild right side tremor	Yes	Unknown	Low dose propranolol; softer mallets; quit snare drum

Lederman 2004	M		51	53	Left	Right	Drum set (Jazz)	Left wrist Flex and ulnar deviation	No	Yes	Limb immobilization trial (unknown long term improvement)

Ragothaman 2004 [11]	M		31	32	Right	Unknown	Tabla	Ext 1, Flex 2, 3, 4, 5, forearm pronation, wrist ulnar deviation	No	No	Botulinum toxin injections (onabotulinumtoxinA)

Ragothaman 2004	M		45	47	Both (R > L)	Unknown	Tabla	Right: wrist Flex and ulnar deviation; Left finger flexion	No	No	Botulinum toxin injections (onabotulinumtoxinA – minimal improvement)

Brandfonbrener 1995 [17]	F		? 19		Left	Right	Unspecified	Left Flex 3, Ext 4, 5	No	Unknown	

Brandfonbrener 1995	M		32		Left	Right	Unspecified	Left Flex 3, loss of control of 4th/5th digits	No	Unknown	

Brandfonbrener 1995	F		25		Right	Right	Unspecified	Right Ext 3, 4, 5	No	Unknown	

Sussman 2015 [18]	Unknown		Unknown		Left	Unknown	Unknown	Left wrist flexion, shoulder abduction	No		

Conti 2008 [19]	M		22		Left	Right	Drums	Tremor	Yes	Unknown	

Rosset-Llobet 2012 [9]	M		23	23	Left leg	Unknown	Drum set (Jazz)	Toe extension, left toe, ankle, knee tension	No	No	Sensory Motor Retraining

Rosset-Llobet 2012	M		20	22	Both legs	Unknown	Drum set (Rock)	Toe flexion, heel elevation	No	No	Modified practice routines

Lee 2014 [8]	M		26	28	Right leg	Unknown	Drum set (Heavy Metal)	Thigh tightness; coactivation of hamstring and quadriceps on EMG	No	No	IncobotulinumtoxinA – slight effect

Katz 2013 [7]	M		45	75	Right leg	Unknown	Drum set	Plantar flexion	No	Yes	AFO; botulinum toxin; Functional Electrical Stimulation of peroneal nerve

Asahi 2018 [20]	M		22	37	Right hand and foot	Left	Drum set	Reported difficulty with control of fine movements in foot; right forearm tightness	No	Yes – writing	Improvement with left Vo thalamotomy

Schirinzi 2018 [21]	M		46	49	Left hand	Right	Unknown	Loss of dexterity in left hand; wrist flexion and internal rotation of forearm	No	Yes	Slight benefit with levodopa (<20%); greater benefit with trihexyphenidyl (6 mg daily) and botulinum toxin injections.

Song 2020 [22]	M		59	59	Left arm	Unknown	Janggu (traditional Korean drum)	Left arm and wrist flexion	No	Yes, in other tasks involving flexion of left arm	Partial benefit with botulinum toxin injections


Abbreviations: DIP (distal interphalangeal); Ext (Extension); F (female); Flex (Flexion); M (male); MCP (metacarpophalangeal); PIP (proximal interphalangeal). Upper extremity digits represented by numbers 1 (thumb) to 5.

All 12 patients with drummers’ dystonia were male. Mean age of dystonia onset was 35.2 ± 10 years, spanning a range of 19 to 53 years. The mean time from symptom onset to diagnosis was 4.1 ± 2.78 years. Nearly all drummers experienced marked professional impairment. Two patients changed careers from musical performance to non-performance focuses, including music education and composition. Information regarding style of music played was obtained in 9 drummers: three were primarily classical percussionists, two played traditional Indian tabla, one played jazz and rock, one played pop, one played African-Cuban and African-Caribbean drums, and two played multiple styles. Information regarding age at which the instrument was first started was available for 7 drummers, with a mean age of 11.8 ± 6.7 years, and a range of 2.5–24 years. Only one drummer, who had a father with writer’s cramp, reported a family history of dystonia. None of the patients in our cohort had genetic testing for variants in dystonia associated genes.

### Clinical features

Only upper limbs were involved in our cohort of drummers: six had left upper limb involvement, five had right upper limb involvement, and one had bilateral involvement. Wrist involvement alone was reported in two patients, and isolated finger involvement in four. Six patients had combined finger and wrist dystonia during drumming. Only one drummer had involvement of any arm region proximal to the wrist, with involuntary shoulder elevation in conjunction with wrist and finger posturing. The pattern of involuntary finger movements was divided between flexion in some drummers and extension in others, though two had a pattern of combined distal finger extension and proximal flexion at the metacarpophalangeal joints. One of the drummers experienced irregular tremor in the left upper extremity when playing with very slow strokes; tremor consisted of irregular flexion/extension movements of the wrist at times intermixed with forearm pronation/supination. Four drummers experienced tremor in their affected hand when engaged in tasks other than drumming.

Four drummers identified specific musical patterns or settings in which the dystonia was most intrusive or severe. For one drummer, this included a single roll, as opposed to double roll strokes. Another patient, a classical Indian tabla player, found that the strokes involving his right index finger were the most difficult to execute, but was able to continue to play passages using the wrist without difficulty. Another patient who also played Indian tabla, but played other styles as well, experienced no dystonia when using mallets, but developed marked dystonic flexion of the right index finger when playing tabla and striking the drum directly with his hand. One drummer found that his dystonia, consisting of thumb, index, and middle finger flexion, was most severe when playing soft passages or when playing the snare drum in particular (***Table 1***, No. 12).

A sensory trick was identified by four of the drummers. One had improvement of dystonic ulnar deviation of the wrist when he rested his right forearm on his right knee or when someone else applied moderate pressure to his right arm. One found that using heavier drum sticks with thinner grips was helpful, while another noted the opposite, with improvement in drumming with thicker drum sticks. The fourth had marked improvement in dystonic flexion of his right index finger by bringing his thumb next to the finger; he also experienced improvement by taping the dystonic finger and from wearing an orthopedic finger splint, and this became his main therapeutic approach to improve the dystonia while drumming. Several patients modified their technique to improve their playing. These modifications included altering the angle of upper limb approach to the drum, changing the angle of drums, or using compensatory postures (e.g., adduction of the left elbow in one drummer so it was closer to his trunk and supination of the forearm in another drummer). Half the drummers (6/12) eventually had spread of dystonia to activities other than drumming. The non-musical tasks that were affected included typing, putting on glasses, drinking from a cup, golfing, manipulating cutlery, writing, and brushing teeth. The other half retained task specificity, with occurrence of dystonic movements triggered exclusively by drumming.

Only one patient identified a definite physical change or medical issue prior to dystonia onset, undergoing a C4–6 cervical fusion four months prior to developing dystonia. Another had EMG findings of chronic denervation/reinnervation in FCU and FDP III/IV on the side of dystonia, suggesting the presence of an ulnar neuropathy. However, he had no detectable weakness in the affected hand or arm, no sensory changes, and had not experienced clinical symptoms suggestive of ulnar neuropathy. Another drummer noted the prior use of a very heavy instrument strapped to his left shoulder, the side on which he developed dystonia, although this was not clearly linked temporally to dystonia symptom onset.

### Interventions

Nearly all drummers had tried a number of interventions in hopes of symptom improvement; most treatment interventions tried were pharmacological. Three patients had trials of carbidopa/levodopa without benefit; one of these three also tried trihexyphenidyl but stopped due to dry mouth. Two other patients tried trihexyphenidyl with one experiencing only mild benefit, and one with no benefit. Four patients tried benzodiazepines, which produced mixed results; diazepam gave some improvement in one patient, but lorazepam gave no benefit to another and clonazepam no clear benefit to two others. One drummer tried baclofen without improvement. One patient reported modest improvement in tremor that accompanied the dystonic posturing with the use of propranolol long-acting 60 mg/day. Seven patients received botulinum toxin injections; others were offered a trial of injections but declined or had injections performed but were lost to follow up. Of those receiving injections, one was noted to have good benefit, one reported 60–70% improvement, another had mild improvement in symptoms but experienced problematic weakness, and one had no benefit. Three patients had botulinum toxin injections performed by other practitioners and information regarding effectiveness of these injections was not available. Other intervention trials included limb immobilization in one drummer, which led to transient weakness and no benefit. Another patient tried physical therapy, massage, and stretching without benefit in addition to several alternative treatments, including laser treatment and magnetic therapy, which were all ineffective.

### Selected Cases and Videos

#### Case 1. (*Table 1*. No. 1; *Video segment 1*)

**Video 1:** Clinical features of drummers’ dystonia in cases 1–4.

A 27-year-old professional musician was evaluated for hand dystonia that developed 4 years previously. Dystonia while playing his drums and xylophone consisted of extension of left 2^nd^–5^th^ digits. With his left hand outstretched when not playing, he had mild ulnar deviation of the left hand and mild tremor. He also had dystonia when typing on a small keyboard. A trial of carbidopa/levodopa resulted in no improvement. He was able to remain professionally involved in music, but not as a performer.

#### Case 2. (*Table 1*. No. 2; *Video segment 2*)

A 44-year-old professional drummer of popular music developed dystonia at age 41. While playing, his right wrist would have involuntary ulnar deviation followed by flexion of all fingers resulting in a curled position. He became unable to move his wrist with involuntary contraction of flexor carpi ulnaris and was then unable to drum with his right hand. He experienced spread of the dystonia to other tasks, including golfing, brushing his teeth, and holding a knife.

#### Case 3. (*Table 1*. No. 3; *Video segment 3*)

A 45-year-old percussionist and teacher first developed dystonia at age 43. He was trained in classical percussion, but in the prior 20 years played primarily Indian tabla. He first developed dystonia during a period of intensive tabla playing in India, in which he played 8–10 hours daily. The dystonic pattern was of involuntary flexion of the right index finger and would occur whenever he used the finger in tabla playing or with other percussive techniques in which the finger was primarily involved. In contrast, he experienced no abnormal postures when using a mallet. Faster passages would reliably trigger the dystonia. He had a clear sensory trick, in which approximating the right thumb to the index finger would dramatically improve the dystonia. In addition to this classic sensory trick, he also identified an external sensory trick in which an orthopedic finger splint or application of tape to the distal affected finger significantly reduced the unwanted postures.

#### Case 4. (*Table 1*. No. 4; *Video segment 4*)

A 25-year-old classical percussionist was evaluated for progressive difficulty controlling the left wrist and fingers while drumming, first evident at age 22. Two years into his symptoms, he developed intermittent, irregular tremor of the left hand while playing, brought on when flexing the wrist. He additionally experienced involuntary dystonic flexion of left 3^rd^–5^th^ digits at the distal and proximal interphalangeal joints while drumming. He had significant difficulty controlling wrist flexion and extension during strokes and had the sense that the wrist was flexing involuntarily with radial deviation. He finished a Master’s degree in performance, but was unable to continue his performance career because of the dystonia.

## Discussion

This largest series of drummers’ dystonia reported highlights the clinical features of this relatively uncommon type of musicians’ dystonia, the results of attempted treatments, and outcomes in 12 patients. These cases span diverse musical styles and techniques of drumming and broaden the spectrum of described phenomenology in drummers’ dystonia. All drummers in our series were male and there was frequent involvement of proximal fingers and the wrist. Only one patient had dystonia proximal to the wrist, consisting of shoulder elevation while playing. The majority had eventual spread of dystonia to tasks other than drumming, and most therapeutic interventions did not yield satisfactory results with several musicians abandoning their performance careers.

There is one previously published series of drummers’ dystonia describing a cohort of 6 percussionists that, similar to our series, was almost exclusively male (5/6) [6]. Median age of onset was 34 years. All but one drummer had predominant or exclusive involvement of the non-dominant arm. The pattern of dystonia was wrist flexion in four, wrist extension in one, and forearm supination in three. Two drummers had jerking or tremor of the hand or arm. Two had involvement of a thumb, but none had involvement of other digits. One patient was able to continue playing by giving up the snare drum and concentrating on percussion instruments using mallets rather than sticks. Three patients tried botulinum toxin injections, but none had lasting benefit and no more than two treatment sessions were administered in each case. One drummer reported significant benefit from trihexyphenidyl taken before each performance. Another patient reported benefit from a trial of limb-immobilization which had been started just prior to the report, but long-term outcomes for this intervention were not available.

Concordant with results of the above study, we also found frequent involvement of the wrist, seen in 8/12 drummers. This propensity for wrist involvement in drummers’ dystonia may reflect the relative frequency and importance of wrist movements in drumming technique [10]. A study evaluating muscle activation in drummers highlighted the centrality of wrist movements in these instrumentalists. Drummers studied noticed that muscle groups producing movement at the wrist were the most important for high-speed movements in their playing, and this was confirmed by objective evaluation [10].

In contrast to the report by Lederman, we found involvement of fingers in nearly all patients in our cohort (10/12). As drumming involves heterogeneous techniques, it is possible that drummers in our cohort utilized techniques or played styles that involve finger movements more than those in the Lederman cohort. At least three patients in our series played styles (i.e., tabla and Cuban-African drumming) that emphasize striking drums directly with the hand more than those that rely on use of a stick or mallet. A similar pattern of dystonia, with wrist and proximal finger involvement, was seen in 2 tabla players with dystonia in a previous report [11]. However, in addition to the tabla and African-Cuban drummers in our cohort, six other players had dystonia involving fingers while playing in styles that generally utilize a stick or mallet. Thus, this dystonic finger pattern does not appear to be exclusive to drummers that strike the drum directly with their hands.

Another notable feature in our cohort was the eventual spread of dystonia to tasks other than drumming in half the patients (6/12). This is similar to the cohort reported by Lederman showing spread to other tasks in 3 of 6 drummers and underscores the importance of inquiring about spread to non-musical tasks and the impact on activities of daily living in musicians’ dystonia patients. The commonality of spread to other activities in the present and prior cohorts is significant as it emphasizes the potential disability over and above occupational impairment, a risk that appears high in this cohort.

The use of the lower extremities in drum-set is somewhat unique among instrumentalists (apart from certain keyboard and organ players,) and presents the potential risk of developing lower extremity dystonia. This has been elsewhere reported in jazz, rock, and heavy metal drummers [8,9], but was not seen in our cohort.

All patients in our series were male, though this may reflect gender selection of the instrument rather than a particular predilection to drummers’ dystonia in men. Male predominance of percussion players has been demonstrated in at least one survey of music students enrolled in German conservatories [12]. Similar findings in this country were observed in a survey administered to all professional symphony players who were members of the International Conference of Symphony and Opera Musicians (ICSOM). The survey documented 93 percussionists, of whom 81 (87%) were male [6]. While men do appear to be over-represented among drummers, the male predominance of our cohort may also reflect the higher incidence, in general, of musicians’ dystonia in men as compared to women, with a ratio as high as 4:1 in some studies [13]. It is difficult to draw conclusions about the relative risk of dystonia among drummers as compared to other instrumentalists. In one evaluation of instrumentalists from eight conservatories [14], 2.8% were percussionists. This percentage falls within the range of proportions of drummers with focal dystonia among all instrumentalists seen with focal dystonia in 4 large case series (1–5%) [3,4,5,6]. These data may suggest that rates of drummers’ dystonia are proportional to drummers’ representation among musicians, but given limited data, more formal assessments and longitudinal follow up of drummers are needed to draw stronger conclusions.

In addition to presenting data from our series, ***Table 1*** also includes 20 previously published cases of drummers’ dystonia from multiple sources, including upper and lower limb dystonia. Similar to our findings, the other drummers are mostly male, play a diversity of drumming styles and techniques, and have frequent involvement of the wrists and fingers. Only four drummers have been reported with lower limb dystonia, and the pattern is variable, including toe flexion, toe extension, plantar flexion/heel elevation, and more diffuse tension in the leg when playing.

The critical importance of reciprocal inhibition of antagonist muscle groups in accurate drumming was demonstrated in a study comparing electromyographic (EMG) activation patterns of healthy drummers and non-drummers in a rapid drumming task [15]. Healthy drummers showed less co-contraction of wrist flexors and extensors compared to non-drummers. In contrast, breakdown in reciprocal inhibition has been demonstrated in EMG studies of drummers with dystonia, though these studies examined lower extremity dystonia in particular [8,9]. A study of accuracy of timing in drummers with upper limb dystonia, however, showed increased variability in timing at fast tempos in dystonic drummers, highlighting the potentially severe impact of dystonia on the fidelity of performance [16].

Strengths of our study include a large cohort of drummers who were evaluated at specialty Movement Disorders clinics by experts in the field, the diversity of drummers and musical styles represented, and detailed clinical and videographic information available. We acknowledge that there are limitations with the study’s retrospective design and that not all information was available for all patients, including detailed evaluations of hours played daily, other details of musical training, and demographic details in some cases. Future prospective studies with clinical, videographic, and other quantitative information such as electrophysiology would be helpful in advancing our understanding of drummers’ dystonia. Additionally, there is a significant need for well-designed clinical trials evaluating the use of botulinum toxin in musicians’ hand dystonia in order to better guide treatment dosing, muscle selection, and injection technique, and to offer better evidence-based data to patients when considering treatment approaches.

## Conclusions

Our large cohort involving drummers who played multiple styles with a variety of techniques showed a pattern of dystonia most commonly involving the wrist and proximal fingers and with a high risk of spread to other tasks. These results, taken in context with previously published reports, support the idea that the drumming style or pattern of movements commonly performed may modulate the risk of a particular region being affected by dystonia. Players that utilize finger movements more frequently as part of their playing have a high risk of finger involvement, though finger involvement was also noted in players utilizing sticks or mallets. Further research is needed in the underlying pathophysiology in order to identify potential environmental strategies to minimize the risk of developing dystonia or to design more effective treatments.
